# Examining the Effects of Netropsin on the Curvature of DNA A-Tracts Using Electrophoresis

**DOI:** 10.3390/molecules26195871

**Published:** 2021-09-28

**Authors:** Jillian Miller, Justin P. Peters

**Affiliations:** Department of Chemistry and Biochemistry, University of Northern Iowa, 1227 West 27th Street, Cedar Falls, IA 50614, USA; millejdk@uni.edu

**Keywords:** A-tract curvature, DNA shape, electrophoresis, netropsin

## Abstract

A-tracts are sequences of repeated adenine bases that, under the proper conditions, are capable of mediating DNA curvature. A-tracts occur naturally in the regulatory regions of many organisms, yet their biological functions are not fully understood. Orienting multiple A-tracts together constructively or destructively in a phase has the potential to create different shapes in the DNA helix axis. One means of detecting these molecular shape differences is from altered DNA mobilities measured using electrophoresis. The small molecule netropsin binds the minor groove of DNA, particularly at AT-rich sequences including A-tracts. Here, we systematically test the hypothesis that netropsin binding eliminates the curvature of A-tracts by measuring the electrophoretic mobilities of seven 98-base pair DNA samples containing different numbers and arrangements of centrally located A-tracts under varying conditions with netropsin. We find that netropsin binding eliminates the mobility difference between the DNA fragments with different A-tract arrangements in a concentration-dependent manner. This work provides evidence for the straightening of A-tracts upon netropsin binding and illustrates an artificial approach to re-sculpt DNA shape.

## 1. Introduction

The sequence-directed curvature of DNA A-tracts in vitro has long been appreciated, yet the biological function of A-tracts in vivo is not fully understood. Macroscopically curved DNA sequences have reduced electrophoretic mobility compared to straight molecules of the same length in polyacrylamide gels [1,2,3,4], even after extrapolation to zero polyacrylamide concentration [5]. Curved DNA molecules were also shown to migrate anomalously slow in free solution [6,7]. Early studies showed that a continuous run of adenine (dA) residues is required for curvature, that the constructive phasing of consecutive A-tracts is needed for an additive effect, and that the number of dA residues is important [8]. Cyclization kinetics experiments with theoretical modeling and computer simulations have determined that A_6_-tracts result in an overall curvature of the DNA helix by 17–21 degrees [9].

The initial observation that A-tracts are straight in crystal structures [10,11] prompted molecular dynamics simulations of A-tracts [12,13,14] and NMR solution structures [15,16], which showed that A-tracts are curved under appropriate solution conditions. Temperature, divalent metal cations, and concentrated monovalent cations have been shown to be important mediators of curvature as well [17,18,19,20]. The degree of the A-tract curvature is also influenced by monovalent cation identity, an effect thought to arise from differential van der Waals radii [21]. The probing of A·T base pair opening kinetics as a function of ammonium cation suggests that the unique A-tract conformation is favored at a lower cation concentration [22].

Chemical modifications also have the ability to modulate curvature; e.g., pyrimidine 5-methyl groups were shown to have a significant effect on the degree of curvature, with a conversion from A_4_T_4_ to the unmethylated counterpart A_4_U_4_ associated with an increase in curvature [23,24]. The mechanism proposed was that methyl groups are modulating the width of the major groove. This further led to a model where sequence-dependent stacking interactions drive the A-tract base pairs into a conformation where the minor groove is narrowed [25]. Bases that prevent this reduction in minor groove width were hypothesized to result in a reduced curvature, e.g., the 2-amino group of guanine or 2-aminoadenine [26,27]. An alternative, electrostatic model argues that a narrow minor groove is the result of the localization of cations and ordered water molecules that form a “spine of hydration” [28,29,30,31,32,33] and that purine 2-amino groups act to disrupt this hydration spine [34]. Similarly, it was argued that the unique hydration pattern, and consequent curvature of DNA A-tracts, can be disrupted by elevating the temperature or adding dehydrating agents [35]. It was also shown that organic cosolvents and dehydrating agents used in crystallization, e.g., 2-methyl-2,4-pentanediol (MPD), decrease the gel mobility anomaly [36,37,38], as well as the rate of cyclization of DNAs that are curved due to phased A-tracts [39,40].

The influence of the flanking DNA sequence has been explored, with changes in flanking residues at most affecting the total curvature by 10–15%. The greatest degree of curvature occurred when the A-tract was flanked by dC on the 5′ side and dT on the 3′ side [41]. High resolution chemical probing revealed that the minor groove of A-tracts progressively narrows from 5′ → 3′ along the dA strand [42]. The surprising result that A_4_T_4_ is curved whereas T_4_A_4_ is not led to “junction” and “wedge” models that neglect thermal fluctuations and deal with static parameters of the DNA base steps [43,44]. It was found that TpA steps, as opposed to ApT steps, have a significantly different local curvature, and that their positioning at the edge of the A-tracts results in an altered global curvature [21,45,46]. This result was confirmed by the differential chemical susceptibility of A_4_T_4_ versus T_4_A_4_ [47].

Evidence that cations bind in the A-tract minor groove [48,49] led to the model of DNA grooves as flexible ionophores that adjust in width in response to cation coordination [50,51]. This model is consistent with the observations that the site-binding of divalent cations, such as Mg^2+^, bridge close phosphate groups, thereby promoting A-tract curvature [52,53]. This model suggests that groove occupancy by site-bound cations determines the observed behavior of A-tract, G-tract, and generic DNA sequences [54,55]. Further studies have shown that monovalent cation binding to the A-tract minor groove likely occurs at most one-third of the time [56,57], and that excess monovalent cation binding to the A-tract minor groove is not the cause of the unique A-tract conformation [20]. These latter studies propose that monovalent cations may be linked to a conformation transition (from B-DNA to so-called B*-DNA or B′-DNA) with the formation of a narrow minor groove, and that specific protein contacts can drive generic DNA sequences into this conformation [58,59].

Antibiotic drugs, such as netropsin, [60,61] preferentially bind at the location of a narrow minor groove of DNA, such as A-tracts, [62,63,64] and directly or indirectly impact the curvature [65]. Netropsin has been shown to form two distinct complexes with the A/T binding sites [66,67], presumably resulting from an equilibrium between the two conformations of the minor groove [68,69]. Flanking bases and cosolutes (such as betaine and MPD) also influence the conformation of the minor groove binding site [70].

Experimentally, we hypothesize that antibiotic drugs with an affinity for A/T sequences, such as netropsin, can straighten DNA A-tracts by binding in the A-tract minor groove. This hypothesis is explored in this work by systematically comparing DNA constructs with in-phase and out-of-phase A-tracts using both gel and capillary electrophoresis. Although polyacrylamide gel electrophoresis (PAGE) is commonly thought to be the most sensitive indicator of DNA curvature, in this work, we demonstrate that very similar mobility differences of the DNA constructs are also observed with capillary electrophoresis (CE). Hence, the A-tract-containing samples exhibit a similar degree of curvature in free solution and in polyacrylamide gels.

## 2. Results and Discussion

**Figure 1:**
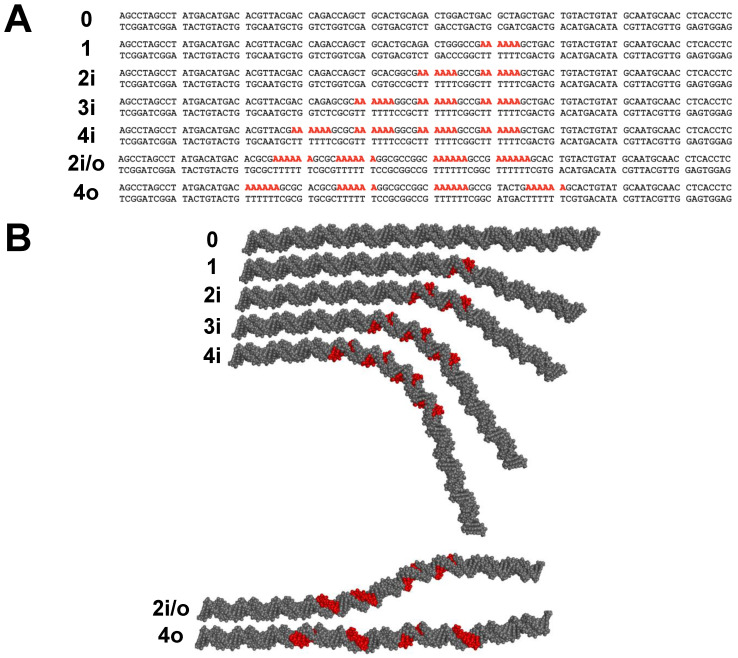
Schematic of 98-bp DNA fragments. (**A**) Sequences of the DNA constructs studied here with A_6_-tracts shown in bold red. Possible unpaired 3′ deoxyadenosine residues from non-template-dependent terminal transferase activity of Taq polymerase are not shown. The first four sequence names (samples 0 to 4i) indicate the number of phased A_6_-tracts in the sample. Sample 2i/o contains two pairs of in-phase A_6_-tracts; the two pairs of phased A_6_-tracts are out-of-phase with each other. Sample 4o has four A_6_-tracts separated by 16 residues from each other. (**B**) Depiction of the shapes of the DNA fragments with A-tracts shown in red. Molecular models were rendered with PyMOL.

Sequences for the 98-bp DNA constructs used in this work are shown in Figure 1A. A-tracts are considered “in-phase” when spaced by an even multiple of the DNA helical repeat, so that the A-tracts all occupy the same face of the DNA double helix and the curvature of the individual A-tracts is additive. Fragments 0, 1, 2i, 3i, and 4i contain the indicated number of in-phase A_6_-tracts. A-tracts can also be spaced by half multiples of the helical repeat, placing them “out-of-phase” on opposite faces of the DNA double helix. Sample 2i/o (in/out) contains two sets of in-phase A_6_-tracts with 15 residues between the first set and second set, making the two pairs out-of-phase with each other. Sample 4o contains four out-of-phase A_6_-tracts. Figure 1B provides a visual representation of the shapes of the DNA constructs imagined in this work.

**Figure 2:**
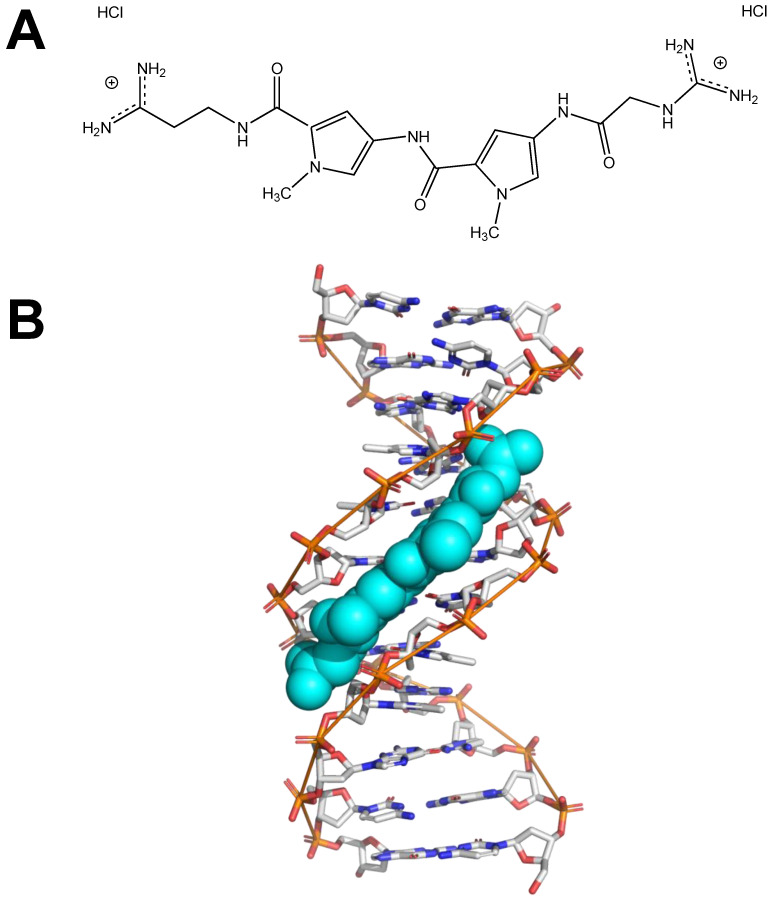
Netropsin. (**A**) Chemical structure of netropsin dihydrochloride, created using ChemDraw. (**B**) Image depicting netropsin (teal spheres) binding the minor groove of DNA (PDB file 121D), created using PyMOL. The hypothesis is that netropsin binding eliminates the curvature of A-tracts.

Netropsin is a small molecule with antiviral and antibiotic properties that preferentially binds AT-rich DNA sequences, including DNA A-tracts [71]. Figure 2A shows the chemical structure of netropsin dihydrochloride. Figure 2B is a visual representation of netropsin (shown in teal spheres) binding the minor groove of an A-tract in a short DNA helix.

### 2.1. Electrophoretic Mobility of DNA in Polyacrylamide Gels

**Figure 3:**
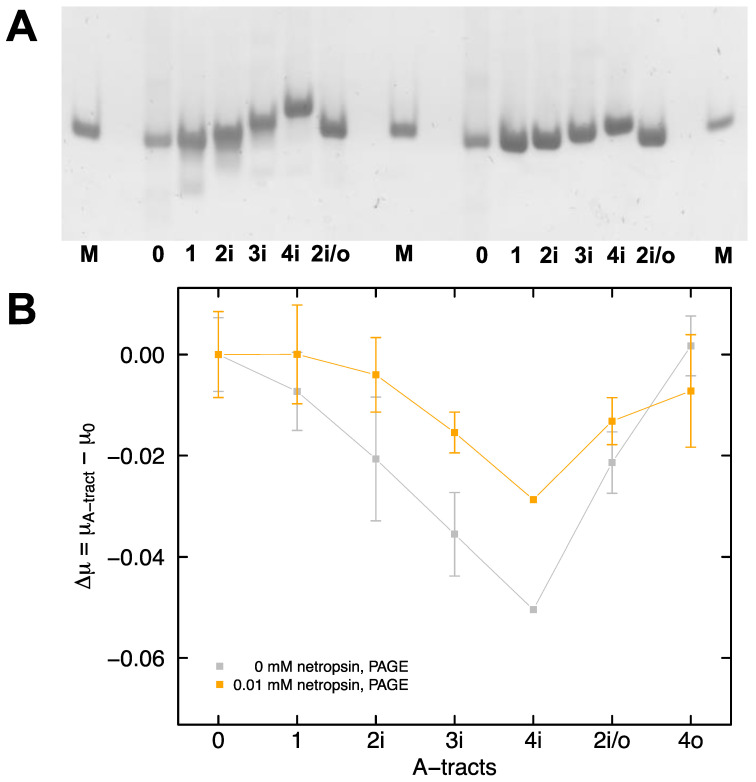
Polyacrylamide gel electrophoresis. (**A**) Representative image of the A-tract samples run in a polyacrylamide gel containing 30 mM Tris^+^ ions without netropsin (left set of bands) or premixed with 0.01 mM netropsin (right set of bands). The various DNA samples are identified at the bottom of each lane. The lanes labeled M correspond to a 100-bp marker. (**B**) The differences in mobility between sample 0, with no A-tracts, and the A-tract-containing samples are plotted as a function of the number and arrangement of the A-tracts in the absence (gray) or presence (orange) of 0.01 mM netropsin. The lines are drawn to guide the eye.

The electrophoretic mobility of DNA in polyacrylamide gels can be calculated from Equation (1) (see Materials and Methods) by measuring the distance the analyte travels in a set amount of time. A representative image, showing the migration of the A-tract samples in polyacrylamide gels cast and run in 0.5X TBE buffer containing 30 mM Tris^+^ ions, is presented in Figure 3A (left set of bands are without netropsin and right set of bands are pre-mixed with 0.01 mM netropsin). The mobilities of samples 1, 2i, 3i, and 4i decrease progressively with an increasing number of phased A-tracts, as expected from previous studies in the literature [20,72]. The mobility of fragment 4i, with four in-phase A-tracts, is significantly reduced compared to that of fragment 2i/o, indicating that mobility is determined by the arrangement of the A-tracts in the sample, and not the number of A-tracts. Samples pre-mixed with 0.01 mM netropsin display a marked diminution of the observed mobility decrease with in-phase A-tracts (Figure 3A, right set of bands). The mobilities observed for the various A-tract constructs in polyacrylamide gels are compared by calculating the difference in mobility between fragment 0, with no A-tracts, and the fragments with one or more A-tracts (Figure 3B). Since the mobilities observed for the in-phase A-tract constructs are smaller than those observed for fragment 0, the mobility differences ∆μ = μ(A-tract) − μ(0) are negative in sign. For the interested reader, all of the mobilities determined in this work are presented in Supplementary Material Table S1.

### 2.2. Electrophoretic Mobility of DNA in Free Solution

**Figure 4:**
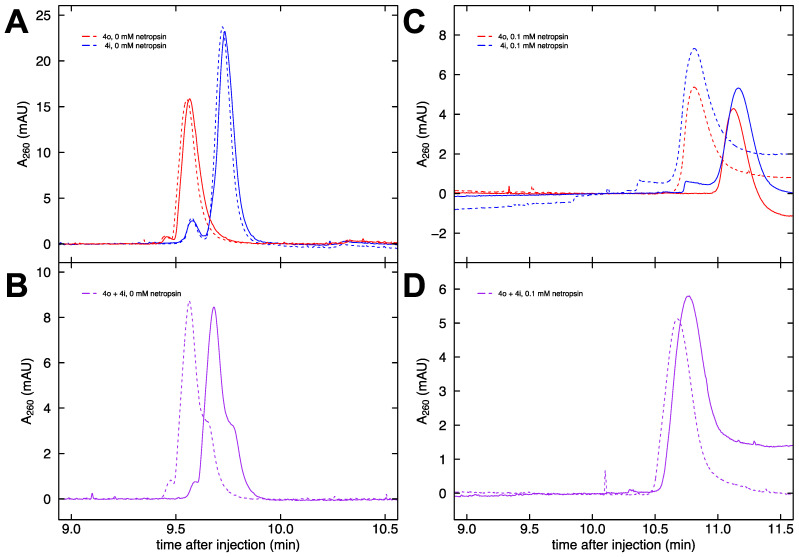
Electropherograms of DNA in free solution. (**A**) Electropherograms of separate runs of fragments 4o (red) and 4i (blue) in buffer containing 33 mM Na^+^ ions in the absence of netropsin. (**B**) Electropherograms of the co-injection of 4o and 4i in buffer containing 33 mM Na^+^ ions in the absence of netropsin. (**C**) Electropherograms of separate runs of fragments 4o (red) and 4i (blue) in buffer containing 33 mM Na^+^ ions and 0.1 mM netropsin. (**D**) Electropherograms of the co-injection of 4o and 4i in buffer containing 33 mM Na^+^ ions and 0.1 mM netropsin.

The apparent electrophoretic mobility of DNA in free solution can be calculated from Equation (1) by measuring the migration time for an analyte to travel a set distance (41.5 cm for the capillaries used in this work). In capillary electrophoresis, the apparent mobility is the sum of the true electrophoretic mobility of the analyte and the mobility due to the electroosmotic flow (EOF) of the solvent. We began with fragments 4o and 4i. Each of these constructs contains four A-tracts, either in-phase or out-of-phase. Hence, any cation localization around the A-tract minor groove should be the same for both samples, and any mobility difference can be attributed entirely to differences in shape. Replicate runs of individual injections of 4o (red) and 4i (blue) are shown in Figure 4A. The DNA concentrations of the injected samples (53 ng/μL for 4o and 64 ng/μL for 4i) are predicted to result in a peak area ratio of 0.84:1. The area under the curve values found from numerical integration using Simpson’s rule for the four injections in Figure 4A yield an average area ratio of 0.76:1. This is in reasonable agreement given the approximate nature of the integration method. 

**Figure 5:**
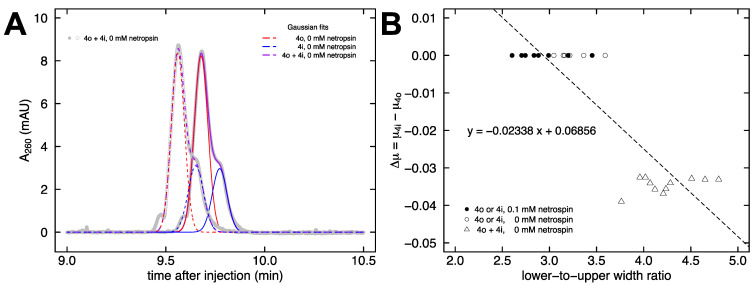
Quantification method. (**A**) The raw data of a co-injection electropherogram (gray) are fit with two Gaussian functions: 4o in red and 4i in blue. The sum of the two Gaussians is in purple. (**B**) Standard curve for the lower-to-upper width ratio method of analysis. The circles represent single injections of 4i or 4o, triangles represent co-injection of 4i with 4o, unfilled symbols represent no netropsin in the buffer, and filled symbols represent 0.1 mM netropsin in the buffer. The equation of the best fit line is given.

To reduce the number of runs performed and to largely eliminate the effects of EOF, the method of co-injection is favored. Samples 4o and 4i were pre-mixed in a concentration ratio of approximately 5:2 for these co-injections (purple), so the raw data in Figure 4B show a pronounced peak (4o) and a smaller shoulder (4i). Specifically, 48 μL of 4o was mixed with 17 μL of 4i, resulting in a concentration ratio of 2.36:1 (with 4i now the lesser component, as intended). The area under the curve values for Figure 4B are calculated from fitted Gaussians (c.f. Figure 5A) and give an average area ratio of 2.43:1, which is in good agreement with the expected ratio based on the concentrations. The difference in retention time from the main peak to the shoulder in Figure 4B is very reproducible since it is largely independent of the effects of EOF. In the absence of netropsin, the mobility difference between the 4o and 4i fragments is comparable between the separate injection and co-injection methods.

Given the variability in EOF between runs, many more replicates are needed to obtain meaningful results from separate injections versus co-injections. Generally, month-to-month variability is greater than the typical variability observed within a given day. This can be seen from the replicate runs (dashed line data collected in a different month than solid line data) shown in Figure 4C of individual injections of 4o (red) and 4i (blue) with the addition of 0.1 mM netropsin to the buffer. However, it is clear from co-injection of 4o and 4i (purple) in the presence of 0.1 mM netropsin that the runs are now characterized by a single peak and that the previously observed mobility difference in the absence of netropsin (Figure 4B) is greatly diminished (Figure 4D). Compared to Figure 4A and Figure 4B, Figure 4C and Figure 4D display a shift in retention times with the addition of netropsin to the buffer. Small single-stranded DNA oligomers that do not interact with netropsin also exhibit this behavior (data not shown). We attribute this result to differences in the relative concentrations, equivalent conductivities, cation sizes, and viscosities from the changing composition of the background electrolyte.

The raw data from Figure 4B, which shows a pronounced peak and a smaller shoulder, can be fit with two Gaussian functions [73]. In Figure 5A, each purple line is the sum of the two fit Gaussian curves (4o in red and 4i in blue), which aligns well with the gray symbols representing the raw data. For the runs with 0.1 mM netropsin added to the buffer, the presence of an observable shoulder in the data is lacking (c.f. Figure 4D), which makes it difficult to apply the Gaussian fit method to obtain quantitative information about any remaining mobility differences. Instead, a new method was devised comparing the full width at 17% and 83% max peak height in the raw data. For an ideal Gaussian curve, the ratio of these full widths (at 0.175699 max relative to 0.824301 max) equals three. This is what is observed for samples 4o and 4i individually in either the presence or absence of netropsin (Figure 5B, circles). As discussed above, the co-injection of both samples together in the absence of netropsin produces a larger peak with a smaller shoulder that can be fit with Gaussians (Figure 5A). The presence of a smaller shoulder below the 83% max height threshold increases the lower-to-upper width ratio to approximately 4.5, since the upper width remains unchanged (Figure 5B, triangles). Figure 5B shows the standard curve that was constructed from these data to allow for the quantification of the mobility difference from co-injections at arbitrary concentrations of netropsin.

### 2.3. Dependence of Free Solution Mobility Difference on Netropsin Concentration

**Figure 6:**
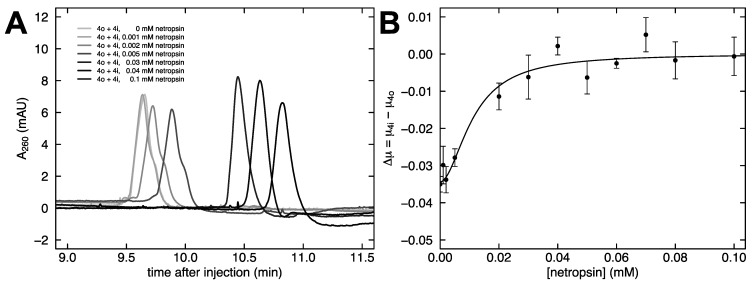
Effect of netropsin concentration. (**A**) Co-injections of 4o and 4i at varying netropsin concentrations from 0 mM netropsin (gray) to 0.1 mM netropsin (black). (**B**) The mobility difference between 4o and 4i at various netropsin concentrations. The curved line corresponds to the fit of a four-parameter sigmoidal function.

The dependence of the free solution mobility difference on netropsin concentration was investigated in more detail. Co-injections of fragments 4o and 4i were performed as the concentration of netropsin in the buffer increased. Figure 6A shows that the distinct feature of a shoulder diminishes with an increasing netropsin concentration. Since the likely cause of the original disparity in mobility between fragments 4o and 4i is shape, we interpret the reversal of this mobility disparity with increasing netropsin concentration as the two fragments converging on a similar shape. In particular, this finding suggests that netropsin binding results in a straightening of the curved fragment 4i.

The relationship between the mobility difference of 4o and 4i and the concentration of netropsin added to the buffer is shown in Figure 6B. A four-parameter sigmoidal function was used to fit the data in Figure 6B. Increasing the netropsin concentration brings the mobility difference to a plateau near zero, and the midpoint of the transition occurs at 0.0112 mM netropsin.

### 2.4. Electrophoretic Mobility of A-Tract Constructs

**Figure 7:**
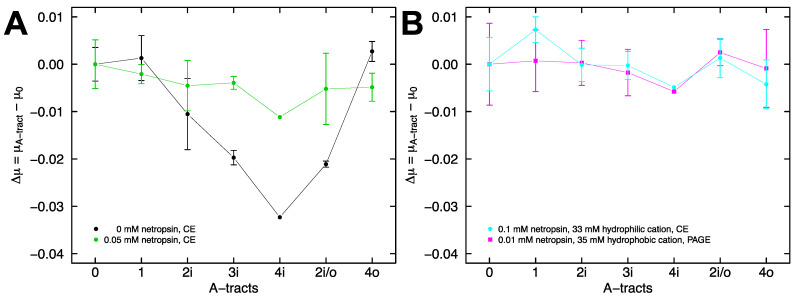
Electrophoresis of A-tract constructs. (**A**) Sodium phosphate buffer, pH 7.28 containing 33 mM Na^+^ ions, was used for all capillary electrophoresis experiments. Plot of the free solution mobility difference between A-tract containing DNA fragments and fragment 0 in the absence (black) or presence (green) of 0.05 mM netropsin. The lines are drawn to guide the eye. (**B**) Free solution mobility difference in the presence of 0.1 mM netropsin (cyan) and gel mobility difference for the combination of 35 mM TBA^+^ ions and 0.01 mM netropsin (magenta). The lines are drawn to guide the eye.

For capillary electrophoresis experiments with the remaining constructs, co-injection of each construct with 4i at the inlet was followed by negative polarity electrophoresis to mobilize the fragments past the detector. All of the fragments exhibited a difference in free solution mobility relative to 4i when electrophoresed in sodium phosphate buffer, pH 7.28 containing 33 mM sodium ions and no netropsin. Figure 7A plots the difference in mobility between each A-tract containing DNA fragment and fragment 0 in the absence of netropsin (black), and the accompanying attenuated mobility difference with addition of 0.05 mM netropsin (green). A similar mobility for all of the DNA fragments in the presence of saturating netropsin, particularly fragments 4i, 2i/o, and 4o, which each contain four A-tracts, implies that they are converging on a similar shape, since shape is the only reasonable explanation for the differences in mobility observed in the absence of netropsin. This result in free solution mirrors the result observed during gel electrophoresis (Figure 3B).

The difference in free solution mobility between fragment 0 and the A-tract constructs is almost completely eliminated in the presence of 0.1 mM netropsin (Figure 7B, cyan). We interpret this result as netropsin binding driving all of the DNA fragments into straight conformations. We have previously shown that A-tract curvature is also lost in solutions containing moderate concentrations of hydrophobic cations, possibly because the bulky tetrabutylammonium ions (TBA^+^) cannot organize as well around the narrow A-tract minor groove [20]. Since netropsin and TBA^+^ each individually promote a straighter A-tract, we sought to investigate the combined effects. We found that, independent of the number of A-tracts and their arrangement in the DNA fragments, the mobility difference observed in polyacrylamide gels run in tetrabutylammonium cacodylate buffer, pH 6.6 (containing 35 mM TBA^+^ ions) is largely absent when 0.01 mM netropsin is added to the buffer (Figure 7B, magenta). Assessed individually, these concentrations are unable to eliminate the A-tract curvature. Therefore, netropsin appears to function synergistically with TBA ions in reducing the A-tract curvature.

## 3. Materials and Methods

### 3.1. DNA Fragments

The DNA fragments in this work were synthesized by polymerase chain reaction (PCR) from plasmids previously described [20]. Briefly, PCR reactions (100 μL) included 20 ng plasmid template, 0.4 μM UNI1 (5′-AGCCTAGCCTATGACATGAC) and 0.4 μM UNI2 (5′-GAGGTGAGGTTGCATTGCAT) (Integrated DNA Technologies, Coralville, IA, USA), 100 μg/mL BSA, Taq DNA polymerase buffer (Invitrogen, Waltham, MA, USA), 2 mM MgCl_2_, 0.2 mM each dNTP, and 5 U Taq DNA polymerase (Invitrogen). Cycle conditions were 94 °C (3 min), 30 cycles of [94 °C (5 s), 55 °C (15 s), and 72 °C (30 s)], 72 °C (5 min), followed by 4 °C. Following PCR, the DNA samples were purified using a GeneJET PCR Purification Kit (Thermo, Waltham, MA, USA).

### 3.2. Buffers

A 20 mM sodium phosphate buffer, pH 7.28 containing 33 mM sodium ions, was prepared by mixing 13.5 mL of 1 M Na_2_HPO_4_ with 6.5 mL of 1 M NaH_2_PO_4_ and 980 mL of water. Stock buffers of 25X TBE (2.5 M Tris base, 2.75 M boric acid, 0.05 M EDTA, pH 8.3) and 50 mM TBA-cacodylate (50 mM tetrabutylammonium hydroxide and 50 mM cacodylic acid, pH 6.6) were also prepared. Since the Tris base is half ionized at its pKa of 8.07 (25 °C), the concentration of Tris^+^ ions in 0.5X TBE buffer is ~30 mM according to the Henderson–Hasselbalch equation. Similarly, the TBA^+^ concentration in the 50 mM TBA-cacodylate buffer is ~35 mM, since cacodylic acid is half ionized at its pKa of 6.27 (25 °C). To avoid confusion, buffers are described by the concentration of the cation, and not the ionic strength of the solution nor the anion concentration. We previously showed that A-tract curvature is lost with increasing concentration of monovalent cations in the buffer [20]. Since cation concentration impacts the magnitude of the observed A-tract curvature, we used a consistent cation concentration (30–35 mM) in this work to avoid confounding effects from cation concentration.

For capillary electrophoresis, a 1 mM stock of netropsin dihydrochloride (Enzo Life Sciences, Farmingdale, NY, USA) was prepared in buffer and diluted to the indicated final concentration, ranging from 0.0005 mM to 0.1 mM. For gel electrophoresis, the netropsin was added to the DNA sample but not included in the running buffer to conserve resources.

### 3.3. Polyacrylamide Gel Electrophoresis (PAGE)

Polyacrylamide gels containing 5% total acrylamide and 5% bisacrylamide crosslinker were cast in running buffer at room temperature. A mixture of 40% acrylamide and bis-acrylamide solution, 19:1 (BioRad, Hercules, CA, USA) diluted in running buffer with 0.1% (*v/v*) *N,N,N′,N′*-tetramethylethylenediamine (TEMED, Sigma, St. Louis, MO, USA) and 0.1% (*w/v*) freshly dissolved ammonium persulfate (Sigma), was slowly poured between glass plates (14.5 × 22.5 cm) assembled with 1.5-mm spacers. Gels were pre-electrophoresed for 20 min, then DNA samples and 100-bp ladder (Invitrogen) were loaded. Using an electric field strength of 8–12 V/cm, the gels were run until markers had migrated 50–90% the length of the gel. Following electrophoresis, gels were stained with 1X Sybr Green I (Invitrogen) in running buffer for 20 min. Images were then obtained using a Typhoon FLA 7000 (GE Healthcare, Chicago, IL, USA), and migration distances (50 micron/pixel) were measured from these digital images.

### 3.4. Capillary Electrophoresis (CE)

The free solution mobilities of the DNA samples were measured with an Agilent 7100 Capillary Electrophoresis System (Agilent, Santa Clara, CA, USA). All measurements were made in the reverse polarity mode with UV detection at 260 nm using a green interface from Agilent. The capillaries were coated internally with linear polyacrylamide (Polymicro Technologies, Phoenix, AZ, USA) to minimize the electroosmotic flow of the solvent. The capillaries were 50.0 ± 0.2 cm in length (41.5 ± 0.1 cm to the detector), had a 75 ± 3 μm internal diameter, and were mounted in a 20.0 ± 0.1 °C temperature-controlled cassette. Following preconditioning of the capillary with a hydrodynamic flush (at 1379 mbar for 240 s) and applied voltage (−10.1 kV for 60 s), the DNA samples were injected hydrodynamically at 34 mbar (0.5 psi) for 3 s; the sample plug occupied ~0.72% of the capillary length. A voltage of −10.1 kV applied over the 50 cm total length of the capillary produced a field strength of 202 V/cm; the current was always 125 μA or less.

### 3.5. Calculation of Mobility

Gel electrophoresis allows for charged particles to separate based on their charge density, size, and shape as they are driven by an electric field. The pores of a slab gel cause steric hinderance to allow size separation during gel electrophoresis, and multiple samples can be processed in parallel within the same gel. Capillary electrophoresis can be used to measure the free solution mobility of the DNA fragments without some of the physical hindrance found in gel electrophoresis. CE also allows for faster run times and less waste when compared to gel electrophoresis [74]. The apparent mobility (µ_a_, cm^2^ V^−1^ s^−1^) of an analyte, such as a DNA sample, is calculated using Equation (1):

(1) $$\mu_{a}=\frac{L}{E\cdot t}$$

based on the length (*L*, cm) of the capillary from the injection site to the detector or the distance migrated in a gel, the applied electric field strength (*E*, V/cm), and the migration time (*t*, s). All mobilities are presented in mobility units (m.u.), where 1 m.u. = 1 × 10^−4^ cm^2^ V^−1^ s^−1^.

Electroosmotic flow (EOF) creates a solvent front caused by electrostatic wall–solvent interactions and varies between runs. For DNA samples, the CE instrument is run in negative polarity mode (negative electrode at the inlet) and EOF is an opposing force to DNA migration. Knowing the magnitude of mobility due to EOF within a capillary allows for both finding the absolute electrophoretic mobility of the DNA sample and confirming the direction the DNA sample will migrate during electrophoresis. Minimizing the mobility due to EOF allows for faster run times and increased reproducibility between runs [74]. For this experimental work, the mobility due to EOF was kept very low with linear polyacrylamide-coated capillaries [75], but did vary slightly from run to run because of transient changes in capillary coating. The mobility due to EOF was measured in every new capillary and new background electrolyte [76]. Unless otherwise stated, co-injections were used to determine a relative mobility between samples, circumventing the variability of mobility due to EOF between runs. The co-injections contained DNA fragment 4i with each of the other fragments of interest, with 4i intentionally having a lower concentration than the second fragment. The difference in concentration allowed for the identification of the fragments because the magnitude of the UV absorbance signal directly reflects the amount of sample being detected. Fragment 4i was chosen for co-injection since it has the slowest migration time of all of the fragments. Thus, 4i has the largest difference in mobility with the other fragments and allows for the best resolution of differences from the co-injections.

The free solution and gel mobilities of the various DNA samples are reported as differences in mobility (∆μ) between sample 0, with no A-tracts, and the mobility of an A-tract-containing sample, such as sample 3i: ∆μ = μa(3i) − μa(0).

## 4. Conclusions

The biological function of A-tracts is not fully understood. The mobility difference between DNA fragments with the same number of A-tracts but with different orientations was systematically examined in order to study DNA shape. This research has shown that the mobility decrement caused by A-tracts in both gels and free solution is reversed by saturating amounts of the small molecule netropsin. For capillary electrophoresis, the method of co-injection is a useful way to compare the mobilities of DNA fragments, since it accounts for EOF variation between runs. Using co-injections of the A-tract constructs, all fragments had a mobility difference from 4i when netropsin was withheld from the buffer. The addition of netropsin decreased the mobility differences between the fragments. At saturating netropsin concentrations there was essentially no mobility difference between any co-injection. Netropsin reverses the mobility decrement caused by differences in shape through a straightening of DNA A-tracts and functions synergistically with bulky hydrophobic cations. This further expands the potential usefulness of netropsin in therapeutics.

## Data Availability

Not applicable.

## Supplementary Materials

The following are available online: Table S1, table of electrophoretic mobilities observed for the A-tract constructs.

## Sample Availability

Samples of the DNA constructs are available from the authors.
