# Supplementary material for: Examining the Effects of Netropsin on the Curvature of DNA A-Tracts Using Electrophoresis
Source: Molecules. 2021 Sep 28;26(19):5871. doi: 10.3390/molecules26195871 (PMC8510488; doi:10.3390/molecules26195871)
Supplement: Supplementary file 1 [file molecules-26-05871-s001.zip › molecules-1346647-supplementary.pdf]

Supporting Information

Examining the Effects of Netropsin on the Curvature of DNA A-tracts using Electrophoresis

Jillian Miller and Justin P. Peters*

Department of Chemistry and Biochemistry

University of Northern Iowa

1227 West 27th Street

Cedar Falls, IA 50614-0423

*To whom correspondence should be addressed at justin.peters@uni.edu

There is one page of supporting information containing a table of electrophoretic mobilities observed for the A-tract constructs.

Table S1. Polyacrylamide gel (PAGE) mobilities and free solution (CE) mobilities observed for the A-tract samples. All mobilities are given in mobility units (m.u.). The average standard deviation of the measured values was ~0.006 m.u. for the PAGE measurements and ~0.004 m.u. for the CE measurements.

PAGE mobilities

Sample	30 mM $Tris^+$, 0 mM netropsin	30 mM $Tris^+$, 0.01 mM netropsin	35 mM TBA^+, 0.01 mM netropsin
0	1.317 ± 0.007	1.299 ± 0.009	0.941 ± 0.009
1	1.310 ± 0.008	1.299 ± 0.010	0.941 ± 0.006
2i	1.296 ± 0.012	1.295 ± 0.007	0.941 ± 0.005
3i	1.281 ± 0.008	1.283 ± 0.004	0.939 ± 0.005
4i	1.266	1.270	0.935
2i/o	1.295 ± 0.006	1.286 ± 0.005	0.943 ± 0.003
4o	1.319 ± 0.006	1.292 ± 0.011	0.940 ± 0.008

CE mobilities

Sample	33 mM Na^+, 0 mM netropsin	33 mM Na^+, 0.05 mM netropsin	33 mM Na^+, 0.1 mM netropsin
0	3.516 ± 0.004	3.113 ± 0.005	3.027 ± 0.006
1	3.518 ± 0.005	3.111 ± 0.002	3.034 ± 0.003
2i	3.506 ± 0.008	3.109 ± 0.005	3.026 ± 0.004
3i	3.496 ± 0.002	3.109 ± 0.001	3.026 ± 0.003
4i	3.484	3.102	3.022
2i/o	3.495 ± 0.001	3.108 ± 0.008	3.028 ± 0.004
4o	3.519 ± 0.002	3.108 ± 0.003	3.023 ± 0.005

Netropsin concentration	4i	4o	$\Delta\mu$
0 mM	3.484	3.519	–0.035 ± 0.002
0.001 mM	3.391	3.421	–0.030 ± 0.005
0.002 mM	3.354	3.388	–0.034 ± 0.004
0.005 mM	3.299	3.327	–0.028 ± 0.002
0.02 mM	3.173	3.184	–0.011 ± 0.004
0.03 mM	3.179	3.185	–0.006 ± 0.006
0.04 mM	3.123	3.121	+0.002 ± 0.002
0.05 mM	3.102	3.108	–0.006 ± 0.004
0.06 mM	3.088	3.091	–0.002 ± 0.001
0.07 mM	3.068	3.063	+0.005 ± 0.005
0.08 mM	3.017	3.019	–0.002 ± 0.005
0.1 mM	3.022	3.023	–0.001 ± 0.005
